# Sinapic Acid Prevents Hypertension and Cardiovascular Remodeling in Pharmacological Model of Nitric Oxide Inhibited Rats

**DOI:** 10.1371/journal.pone.0115682

**Published:** 2014-12-22

**Authors:** Thangarasu Silambarasan, Jeganathan Manivannan, Mani Krishna Priya, Natarajan Suganya, Suvro Chatterjee, Boobalan Raja

**Affiliations:** 1 Cardiovascular Biology Lab, Department of Biochemistry and Biotechnology, Faculty of Science, Annamalai University, Tamil Nadu, India; 2 Vascular Biology Lab, AU-KBC Research Centre, Anna University, Chennai, Tamil Nadu, India; 3 Department of Biotechnology, Anna University, Chennai, Tamil Nadu, India; Texas A& M University Health Science Center, United States of America

## Abstract

**Objectives:**

Hypertensive heart disease is a constellation of abnormalities that includes cardiac fibrosis in response to elevated blood pressure, systolic and diastolic dysfunction. The present study was undertaken to examine the effect of sinapic acid on high blood pressure and cardiovascular remodeling.

**Methods:**

An experimental hypertensive animal model was induced by L-NAME intake on rats. Sinapic acid (SA) was orally administered at a dose of 10, 20 and 40 mg/kg body weight (b.w.). Blood pressure was measured by tail cuff plethysmography system. Cardiac and vascular function was evaluated by Langendorff isolated heart system and organ bath studies, respectively. Fibrotic remodeling of heart and aorta was assessed by histopathologic analyses. Oxidative stress was measured by biochemical assays. mRNA and protein expressions were assessed by RT-qPCR and western blot, respectively. In order to confirm the protective role of SA on endothelial cells through its antioxidant property, we have utilized the *in vitro* model of H_2_O_2_-induced oxidative stress in EA.hy926 endothelial cells.

**Results:**

Rats with hypertension showed elevated blood pressure, declined myocardial performance associated with myocardial hypertrophy and fibrosis, diminished vascular response, nitric oxide (NO) metabolites level, elevated markers of oxidative stress (TBARS, LOOH), ACE activity, depleted antioxidant system (SOD, CAT, GPx, reduced GSH), aberrant expression of TGF-β, β-MHC, eNOS mRNAs and eNOS protein. Remarkably, SA attenuated high blood pressure, myocardial, vascular dysfunction, cardiac fibrosis, oxidative stress and ACE activity. Level of NO metabolites, antioxidant system, and altered gene expression were also repaired by SA treatment. Results of *in vitro* study showed that, SA protects endothelial cells from oxidative stress and enhance the production of NO in a concentration dependent manner.

**Conclusions:**

Taken together, these results suggest that SA may have beneficial role in the treatment of hypertensive heart disease by attenuating fibrosis and oxidative stress through its antioxidant potential.

## Introduction

High blood pressure or hypertension is the leading preventable risk factor for cardiovascular diseases and is estimated to account for about 54% of deaths from stroke and 47% of deaths from coronary heart disease in adults worldwide [1]. Myocardial remodeling has an important role in the pathophysiology of hypertensive disease [2]. Arterial hypertension, which modifies the structural and functional features of the myocardium and the blood vessels, a process known as cardiovascular (CV) remodeling. Cardiac remodeling involves molecular, cellular, and interstitial changes that manifest clinically as changes in size, shape, and function of the heart after injury or stress stimulation [3]. Vascular remodeling comprise of structural changes of the arterial walls, such as increased intima-media thickness, arterial stiffening, and deteriorating endothelial function [4].

Cardiac hypertrophy is a common type of cardiac remodeling that occurs when the heart experiences elevated workload. Pathological cardiac hypertrophy involves cellular and molecular remodeling such as myocyte growth without significant proliferation and alterations in the expression of proteins involved in excitation-contraction coupling [5]. Myocardial fibrosis is a common pathological feature seen in many patients with hypertension and is hypothesized to be the final common pathway that ultimately results in irreversible heart failure [6].

Oxidative stress occurs when there is an imbalance between the generation of reactive oxygen species (ROS) and the antioxidant defense systems so that the latter become overwhelmed [7], [8]. ROS stimulates myocardial growth, matrix remodeling, and cellular dysfunction. It also activates a broad variety of hypertrophy signaling kinases and transcription factors [9]. ROS are produced by all vascular cell types, including endothelial, smooth muscle, and adventitial cells, and can be formed by numerous enzymes [10]. Physiologically, NADPH oxidase-derived ROS have been implicated in the regulation of vascular tone by modulating vasodilation directly or indirectly by decreasing nitric oxide bioavailability through quenching by O_2_ **^.^** ^–^ to form ONOO^−^ [11].

Nitric oxide (NO) helps to maintain vascular tone, inhibits endothelial cell stimulation, and is a regulator of platelet activation [12], [13]. Chronic inhibition of NO produces volume-dependent elevation of blood pressure [14]. Bioavailability of NO can be maintained by inhibition of oxidative stress, and therefore the agents with antioxidant properties inactivating free radicals increase NO bioavailability and can improve regulation of vascular tone [15]. In recent years, much attention has been focused on the protective properties of exogenous antioxidants in biological systems, and on the mechanisms of their action. Sinapic acid (SA), a phenolic acid is a cinnamic acid derivative, which possesses 3,5-dimethoxyl and 4-hydroxyl substitutions in the phenyl group of cinnamic acid. It is widely distributed in the plant kingdom and is obtained from various sources such as rye, fruits and vegetables [16]. It has already been pharmacologically evaluated for its potent antioxidant [17], [18], antihyperglycemic [19] and peroxynitrite scavenging effects [20]. In the light of its positive effects, we hypothesized that SA could also have beneficial effects in hypertension. Therefore, the aim of the present study was to investigate the effect of SA on high blood pressure and cardiovascular remodeling in pharmacological model of nitric oxide inhibited rats.

## Materials and Methods

### Animals and chemicals

Male albino Wistar rats, 8-10 weeks old weighing 180–220 g were obtained from the Central Animal House, Annamalai University, India. The animals housed three to a polypropylene cage were provided with standard pellet diet (Kamadhenu Agencies, Bengaluru, India) and water *ad libitum* and maintained under controlled conditions of temperature and humidity with an alternating light/dark cycle in accordance with the guidelines of the Committee for the Purpose of Control and Supervision of Experiments on Animals, New Delhi, India and approved by the Animal Ethical Committee of Annamalai University (approval no: 926).

Nω-Nitro-L-arginine methyl ester hydrochloride (L-NAME) and sinapic acid (SA) were purchased from Sigma-Aldrich (St. Louis, Missouri, USA). All other chemicals used in this study were of analytical grade obtained from Merck and Himedia, India.

### L-NAME hypertensive animal model and sinapic acid treatment

Animals were given L-NAME in drinking water at a dosage of 40 mg/kg body weight (b.w.) for 4 weeks [21]. SA was dissolved in corn oil (vehicle – 5 mL/kg) and administered orally everyday using an intragastric tube for a period of 4 weeks.

Each of the following groups consisted of six animals. Group I: Control; Group II: Rats were treated with SA (40 mg/kg b.w.); Group III: Rats were given L-NAME (40 mg/kg b.w.); Group IV: Rats were simultaneously treated with L-NAME (40 mg/kg b.w.) and SA (10 mg/kg b.w.); Group V: Rats were simultaneously treated with L-NAME (40 mg/kg b.w.) and SA (20 mg/kg b.w.); Group VI: Rats were simultaneously treated with L-NAME (40 mg/kg b.w.) and SA (40 mg/kg b.w.). Vehicle was administered alone to control (Group I) and L-NAME control rats (Group III) orally using an intragastric tube daily for 4 weeks. Body weight was measured daily for all rats.

### Blood pressure measurement

Before commencement of the experiment, animals were trained with instrument for measuring blood pressure. In all groups of animals, systolic blood pressure (SBP) was measured every week during the entire period of the study noninvasively using a tail cuff method (IITC, model 31, USA). Values reported are the average of three sequential blood pressure measurements.

### Heart weight and collagen content

The heart was dissected out and then weighed. Heart weight-to-body weight ratio was calculated. As an estimate of collagen content, hydroxyproline concentration was determined in left ventricular samples as described previously [22]. Hydroxyproline concentration was determined from standard curve and expressed as mg/g dry weight.

### Histopathology of heart and aorta

Excised heart and aorta samples were cleared of blood and immediately fixed in 10% formalin. 5 µm thick tissue sections from heart and aorta were prepared from processed paraffin-embedded samples. For the histological examination of the collagen accumulation, masson's trichrome and picrosirius red staining were used. Heart sections were stained with picrosirius red and masson's trichrome stains, aortic sections were stained with masson's trichrome and examined under a light microscope for evidence of fibrotic changes in tissues.

### Antioxidants and lipid peroxidation

Heart and aortic tissues were sliced into pieces and homogenized in 0.1 M Tris-HCl buffer in cold condition (pH 7.4) to give 20% homogenate (w/v). The homogenate was centrifuged at 560 × g for 10 min at 4°C. The supernatant was separated and used for various biochemical estimations.

Superoxide dismutase (SOD) activity was assayed in the tissues (heart and aorta) by the method of Kakkar et al. [23]. The assay mixture contained 1.2 mL of sodium pyrophosphate buffer, 0.1 mL of phenazine methosulphate, and 0.3 mL of nitroblue tetrazolium and appropriately diluted enzyme preparation in a total volume of 3 mL. The reaction was started by the addition of 0.2 mL of nicotinamide adenine dinucleotide (NADH). After incubation at 30°C for 90 s, the reaction was arrested by the addition of 1.0 mL of glacial acetic acid. The reaction mixture was stirred vigorously and shaken with 4.0 mL of n-butanol. The mixture was allowed to stand for 10 min; centrifuged and n-butanol layer was separated. The color density of the chromogen in n-butanol was measured at 510 nm against butanol blank.

The activity of catalase in the tissues was assayed by the method of Sinha [24]. To 0.9 mL of phosphate buffer, 0.1 mL of tissue homogenate and 0.4 mL of H_2_O_2_ were added. The reaction was arrested after 60 s by adding 2.0 mL of dichromate–acetic acid mixture. The tubes were kept in a boiling water bath for 10 min and the color developed was read at 620 nm.

The activity of glutathione peroxidase (GPx) in the tissues was measured by the method of Rotruck et al. [25]. To 0.2 mL of Tris buffer, 0.2 mL of ethylene diamine tetraacetic acid (EDTA), 0.1 mL of sodium azide, and 0.5 mL of tissue homogenate were added. To the mixture, 0.2 mL of glutathione followed by 0.1 mL of H_2_O_2_ was added. The contents were mixed well and incubated at 37°C for 10 min along with a tube containing all reagents except the sample. After 10 min, the reaction was arrested by the addition of 0.5 mL of 10% TCA. The tubes were centrifuged and the supernatant was used for the estimation of glutathione.

Reduced glutathione (GSH) in the tissues was estimated by the method of Ellman [26]. 0.5 mL of homogenate was pipetted out and precipitated with 2.0 mL of 5% TCA. A total of 2.0 mL of supernatant was taken after centrifugation and 1.0 mL of Ellman's reagent and 4.0 mL of 0.3 M disodium hydrogen phosphate were added. The yellow color developed was read at 412 nm. Total protein was assayed by the method of Lowry et al. [27].

The level of thiobarbituric acid reactive substances (TBARS) in tissues was estimated by the method of Niehaus and Samuelson [28]. A total of 0.5 mL of tissue homogenate was diluted with 0.5 mL of double distilled water and mixed well, and then 2.0 mL of thiobarbituric acid (TBA)–trichloroacetic acid (TCA)–hydrochloric acid (HCL) reagent was added. The mixture was kept in boiling water bath for 15 min. After cooling, the tubes were centrifuged for 10 min and the supernatant was taken for measurement. The absorbance was read at 535 nm against reagent blank.

Estimation of tissue lipid hydroperoxides (LOOH) was done by the method of Jiang et al. [29]. Fox reagent (0.9 mL) was mixed with 0.1 mL of tissue homogenate and incubated for 30 min at room temperature. The color developed was read at 560 nm.

### Aortic nitric oxide metabolites level

Nitric oxide (NO) easily breaks down with the presence of free radicals, hence aortic nitrite levels were measured as a level of NO inactivated due to superoxide radical (O_2–_). Nitrite was estimated colorimetrically with the Griess reagent in aortic homogenate. Briefly equal volumes of aortic homogenate and Griess reagent (sulfanilamide 1% w/v, naphthylethylenediamine dihydrochloride 0.1% w/v, and orthophosphoric acid 2.5% v/v) were mixed and incubated at room temperature for 10 min and the absorbance was determined at 540 nm wavelength [30]. Nitrite was determined from the standard curve obtained using sodium nitrite as standard. The amount of nitrite formed was normalized to the protein content of the respective aorta.

### Estimation of ACE activity

The angiotensin-converting enzyme (ACE) activity in heart and aortic tissues was measured by the spectrophotometric assay as described previously [31]. ACE activity in tissue homogenate was measured by hydrolysis of Hip–His–Leu. Briefly, Hip–His–Leu was hydrolyzed into hippuric acid and His–Leu by ACE. Hippuric acid was extracted by ethyl acetate and determined at 228 nm. ACE activity was expressed as mU per mg protein.

### Langendorff isolated heart study

The left ventricular function of the rat heart was assessed using the Langendorff heart preparation. Briefly, after anesthesia, the heart was excised and placed in cooled (4°C) Krebs Henseleit bicarbonate solution [composition (in mM): 118 NaCl, 4.7 KCl, 1.2 MgSO_4_, 1.2 KH_2_PO_4_, 2.3 CaCl_2_, 25.0 NaHCO_3_, 11.0 glucose]. The heart was then attached to the cannula through aorta and retrogradely perfused with the Krebs solution maintained at 37°C and continuously gassed with a mixture of 95% O_2_–5% CO_2_. Perfusion pressure was kept constant at 80 mmHg. Isovolumetric recordings of rate of pressure development (+dp/dt) and rate of pressure decline (−dp/dt) were obtained from a balloon catheter inserted into the left ventricle. The ventricular balloon was connected via fluid-filled tubing to a pressure transducer (AD Instruments, Australia) for continuous assessment of ventricular performance as described previously [32].

### Isolated aortic ring experiment

The thoracic aorta was dissected out, cleaned of surrounding connective tissue and placed in freshly prepared ice-cold Modified Krebs–Henseleit solution (MKHS) of the following composition (mM): 118 NaCl, 4.7 KCl, 2.5 CaCl_2_, 1.2 MgSO_4_, 11.9 NaHCO_3_, 1.2 KH_2_PO_4_ and 11.1 D-glucose (pH of 7.4). The arterial segments were mounted on two stainless steel hooks and suspended in 10 mL organ baths containing MKHS. Bathing solution was continuously aerated with 95% O_2_–5% CO_2_ mixture and maintained at 37°C. An optimal baseline tone of 1.5 g was applied to all the rings. Relaxations in response to acetylcholine (Ach) and sodium nitroprusside (SNP) were examined in phenylephrine pre-contracted aortic rings. Tension was recorded using a high sensitivity isometric force transducer and stored in a computer using Chart version 6.1.3 software program (Powerlab, AD Instruments, Bella Vista, NSW, Australia) for further analysis. Differences between groups were assessed by ANOVA using Graph Pad Prism version 4 (San Diego, California, USA). Concentration response curves were analyzed using two-way ANOVA followed by Bonferroni tests to compare individual points. Significance was defined as *P*<0.05. All the inducer concentrations were used as described previously [33].

### mRNA expression analysis

After the study period, the heart and aortic tissues were subjected to total RNA extraction using RNA isolation kit (BioUltra). The integrity, quality and quantity of RNA were determined by nano-drop spectrometer. 1 µg of RNA was used for real time quantitative polymerase chain reaction (RT-qPCR) using SYBR green method. SYBR Green JumpStart Taq Ready Mix real time PCR kit (Sigma Aldrich, USA) was utilized as per manufacturer's instructions for reverse transcription and amplification. Primer sequences for forward primer (FP) and reverse primer (RP) were as follows; Transforming growth factor-β (TGF-β) FP: 5′- ATGACATGAACCGACCCTTC-3′ and RP: 5′-GTAGTTGGTATCCAGGGCTCTC-3′; β-myosin heavy chain (β-MHC) FP: 5′-GTAGACAAGGGCAAAGGCAA-3′ and RP: 5′-GGATGATGCAGCGTACAAAG-3′; Endothelial nitric oxide synthase (eNOS) FP: 5′-CTGGCAAGACCGATTACACGA-3′ and RP: 5′-CGCAATGTGAGTCCGAAAATG-3′; glyceraldehydes-3-phosphate dehydrogenase (GAPDH) FP: 5′-ACCACAGTCCATGCCATCAC-3′ and RP: 5′-TCCACCACCCTGTTGCTGTA-3′. The amplification specificity of all the primers was identified through resolving the PCR products by agarose gel electrophoresis. The relative fold change method was employed for calculating the differential expression between samples [34]. Fold change values were averaged from three reactions.

### Protein expression study

Tissue proteins were extracted by homogenization of aortas in Radio-Immunoprecipitation Assay (RIPA) buffer followed by centrifugation at 10,000 × g for 20 min. The supernatants were collected, and protein concentrations were estimated using the BCA protein assay kit (Merck, India). Protein samples were separated with 10% SDS-polyacrylamide gel electrophoresis gels and electrophoretically transferred onto polyvinylidene difluoride (PVDF) membranes (Merck Millipore, USA). Nonspecific binding sites were blocked by 1% BSA in PBS with 0.05% TWEEN 20 at room temperature for 1 hour, then incubated overnight at 4 °C with primary antibody against eNOS (1∶1000 dilution) and then incubated with anti-mouse IgG (Sigma-Aldrich, USA) conjugated to horseradish peroxidase. The reaction was developed with a DAB detection system (Merck, India). The densitometric analysis was performed using ImageJ software (NIH, Bethesda, MD). Western Blot densitometry data of eNOS was normalized to β-actin.

### Endothelial cell culture and *in vitro* oxidative stress study

EA.hy926, an immortalized endothelial hybrid cell line was a kind gift from Dr. C.J.S. Edgell, Mayo Clinic, Rochester, MN, USA. The cells were maintained in DMEM medium containing 10% fetal bovine serum, 1% penicillin/streptomycin at 37°C, 5% CO_2_. Cells that were between passages 4 and 10 were used in this study. All the assays were performed by seeding EA.hy926 cells in the concentration of 1×10^4^ cells/well in 96-well plate. In order to evaluate the cytotoxic effect, EA.hy926 cells were treated with SA (25–100 µM) for 24 h and checked for its viability using MTT assay. For evaluation of protective potential of SA against oxidative stress, H_2_O_2_-induced *in vitro* oxidative stress model was developed as described previously [35]. In this study, different concentrations of SA (0.1, 1 and 10 µM) in DMEM medium were added to the wells and incubated for 24h, then the cells were exposed to 300 µM H_2_O_2_ for further 4h. For viability analysis, MTT solution (1 mg/mL) was added to each well, and the plate was incubated for 4 h at 37°C. After incubation and formazan solubilisation, optical density (OD) of each well was measured on a microplate reader at 570 nm. The OD of formazan formed in untreated cells was taken as 100% viability. We have chosen 10 µM dose of SA for the evaluation of total ROS and NO level. The level of intracellular ROS formation was quantified using redox-sensitive fluorescent probe 2, 7-dichlorodihydrofluorescin diacetate (DCFH-DA). NO level was measured using 0.1 µM of diaminorhodamine-4M (DAR-4M AM) dye and the images were acquired using Olympus IX71 inverted fluorescence microscope as described previously [36]. The fluorescence intensity of the DCF and DAR images was calculated using Adobe Photoshop version 7.0.

### Statistical analysis

Data were analyzed by one-way analysis of variance (ANOVA) followed by Duncan's multiple range test (DMRT) using statistical package for the social science (SPSS) software version 14.0. For aortic relaxation, data were analyzed by two-way ANOVA followed by Bonferroni post hoc test. Values were considered significant when *P*<0.05.

## Results

### Blood pressure and cardiovascular remodeling

**Figure 1:**
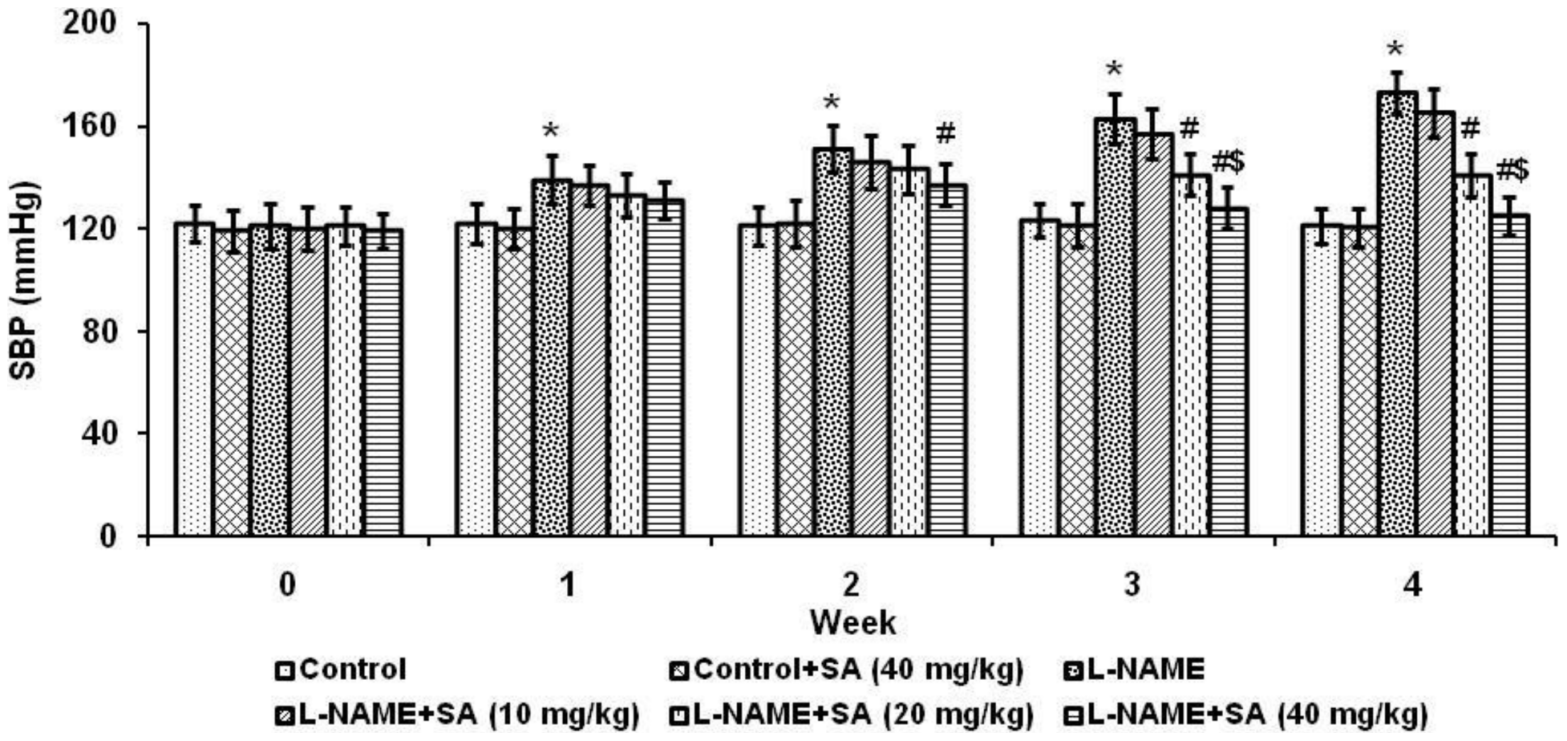
Sinapic acid attenuated increase in systolic blood pressure in experimental hypertensive rats. Values are expressed as mean ± SD. n = 6 per group. ^*^ *P*<0.05 vs control; ^#^ *P*<0.05 vs L-NAME; ^$^ *P*<0.05 vs L-NAME+SA (20 mg/kg).

Fig. 1 shows the effect of sinapic acid (SA) at three different concentrations (10, 20 and 40 mg/kg) on systolic blood pressure. L-NAME treated rats showed significantly (*P*<0.05) increased systolic blood pressure when compared with control and this increase was attenuated by SA treatment. The dosage of 40 mg/kg showed better effect in the reduction of blood pressure compared with other two doses. Hence, 40 mg/kg dose was selected for further evaluation.

**Figure 2:**
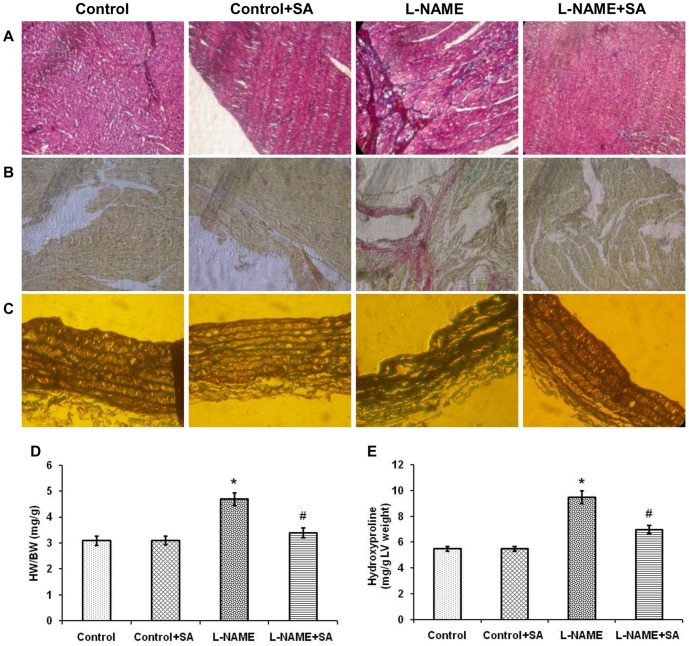
Sinapic acid inhibited cardiovascular remodeling in experimental hypertensive rats. (A) Representative pictures of myocardial tissue sections stained with masson's trichrome. (B) Representative pictures of myocardial tissue sections stained with picrosirius red. (C) Representative pictures of aortic tissue sections stained with masson's trichrome. In all the above three panels (A, B & C) tissue sections from L-NAME group showed fibrosis and this was attenuated in L-NAME+SA group. (D) Estimation of heart weight-to-body weight ratio in various experimental groups. (E) Quantification of hydroxyproline level in various experimental groups. Values are expressed as mean ± SD. n = 6 per group. ^*^ *P*<0.05 vs control; ^#^ *P*<0.05 vs L-NAME.

The heart weight-to-body weight ratio was significantly (*P*<0.05) increased in L-NAME rats and this increase was attenuated by SA treatment (Fig. 2D). L-NAME induced hypertension significantly (*P*<0.05) increased hydroxyproline level when compared with control and this increase was prevented by oral SA treatment (Fig. 2E).

The heart sections with masson's trichrome and picrosirius red staining showed elevated accumulation of collagen and interstitial fibrosis in L-NAME heart whereas SA treatment markedly reduced all the above changes (Fig. 2A and B, respectively). Aortic sections from L-NAME rats with masson's trichrome staining showed marked vascular fibrosis and this change was prevented by administration of SA (Fig. 2C).

### Biochemical variables measured

**Figure 3:**
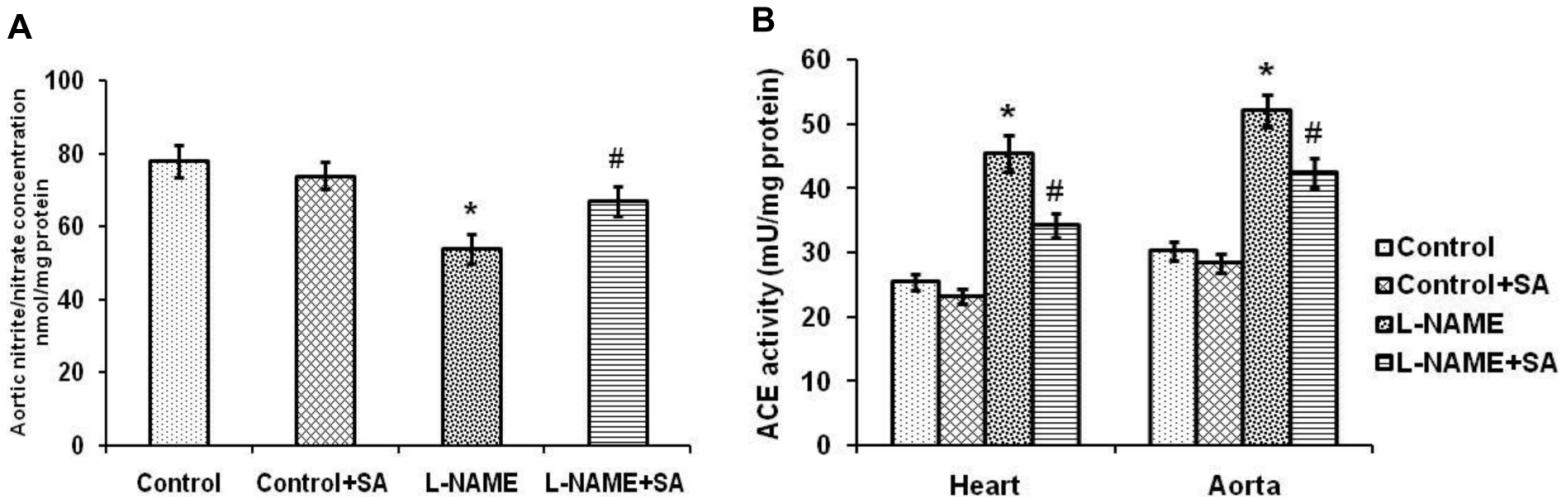
Sinapic acid restores nitric oxide metabolites level and ACE activity in experimental hypertensive rats. (A) Estimation of nitric oxide metabolites level in various experimental groups. (B) Assessment of ACE activity in various experimental groups. Values are expressed as mean ± SD. n = 6 per group. ^*^ *P*<0.05 vs control; ^#^ *P*<0.05 vs L-NAME.

The activities of enzymatic antioxidants such as SOD, CAT, GPx and the level of non-enzymatic antioxidant GSH, lipid peroxidation products such as TBARS, LOOH were presented in Table 1. L-NAME induction significantly (*P*<0.05) reduced the activity of above enzymes, level of GSH and increased the formation of lipid peroxidation products. Treatment with SA significantly (*P*<0.05) restored the activity of enzymatic antioxidants, level of GSH and reduced the level of lipid peroxidation products. Aortic nitrite/nitrate level was significantly (*P*<0.05) reduced in L-NAME rats whereas SA treatment for 4 weeks significantly (*P*<0.05) restored the above (Fig. 3A). L-NAME hypertension significantly (*P*<0.05) enhanced the activity of ACE in heart and aorta compared with control and this increase was attenuated by SA treatment (Fig. 3B).

**Table 1:** Sinapic acid prevented oxidative stress.

Parameter	Sample	Control	Control+SA	L-NAME	L-NAME+SA
SOD (U*/mg protein)	Heart	7.11±0.36	7.02±0.31	3.67±0.29*	5.42±0.41#
	Aorta	12.33±0.82	12.79±0.78	7.61±0.43*	10.18±0.72#
CAT (U#/mg protein)	Heart	49.23±3.56	50.11±2.85	32.28±2.86*	43.18±3.41#
	Aorta	55.6±3.24	56.21±2.73	35.44±2.67*	47.22±3.55#
GPx (U^$^/mg protein)	Heart	6.89±0.38	6.45±0.33	4.18±0.35*	5.29±0.28#
	Aorta	8.18±0.51	8.29±0.45	5.12±0.43*	7.05±0.38#
GSH (μg/mg protein)	Heart	8.68±0.48	8.53±0.42	5.12±0.47*	7.06±0.39#
	Aorta	7.45±0.38	7.33±0.27	4.56±0.35*	6.24±0.39#
TBARS (mmol/100 g wet tissue)	Heart	0.61±0.03	0.58±0.02	2.57±0.15*	0.96±0.04#
	Aorta	0.53±0.03	0.51±0.02	1.81± 0.09*	0.75±0.03#
LOOH (mmol/100 g wet tissue)	Heart	62.34±4.16	60.46±3.79	123.44±9.23*	79.56±4.19#
	Aorta	74.27±5.11	71.29±4.56	108.23±8.17*	84.56±5.22#

U^*^  =  enzyme concentration required to inhibit the chromogen produced by 50% in one minute under standard condition. U^#^  =  μM of H_2_O_2_ consumed/minute. U^$^  =  μg of GSH utilized/minute. Values are expressed as mean ± SD.

^*^ *P*<0.05 vs control;

# *P*<0.05 vs L-NAME; n  =  6 per group.

### Cardiovascular function – Langendorff and organ bath study

**Figure 4:**
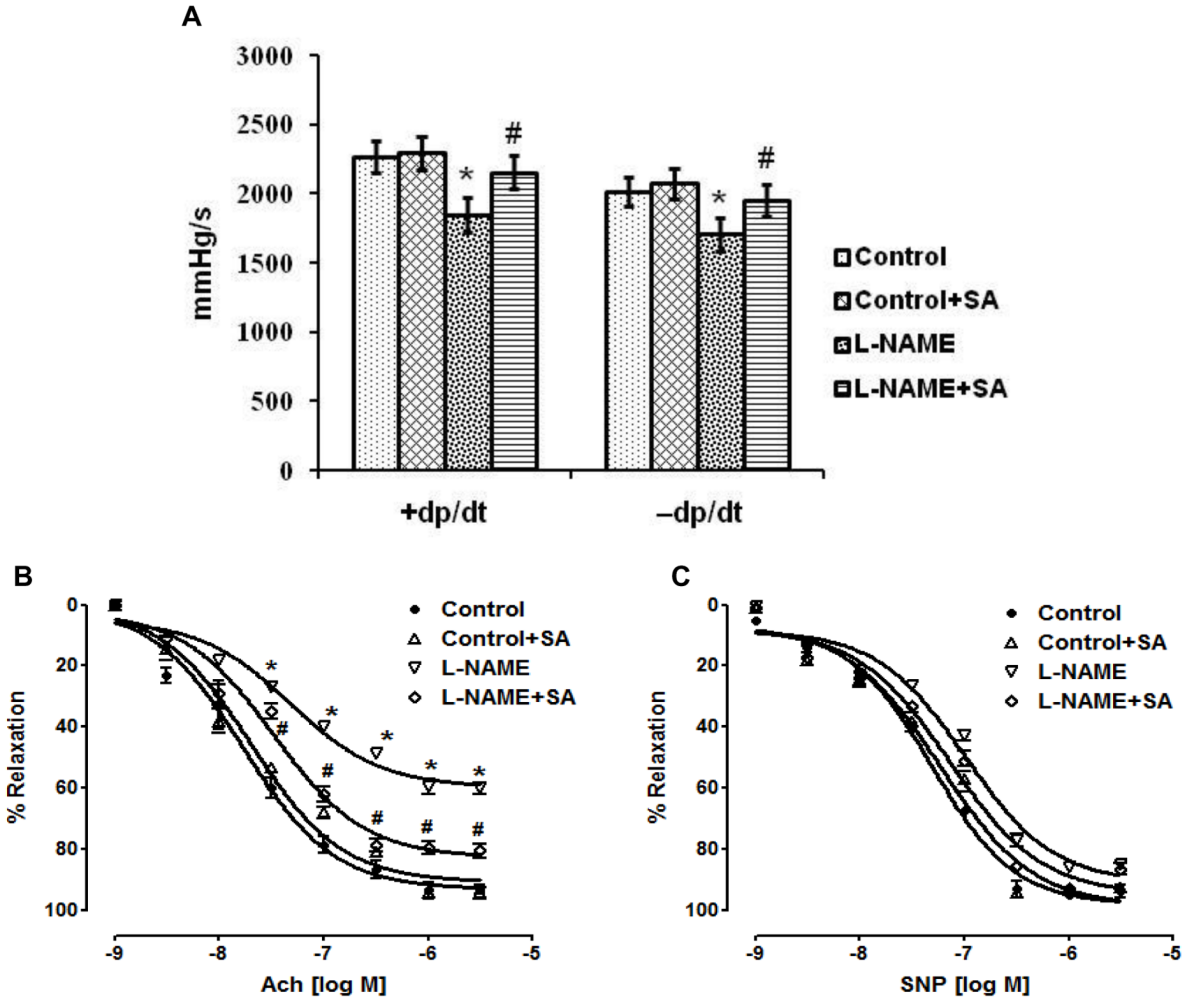
Sinapic acid improved cardiovascular function in experimental hypertensive rats. (A) Evaluation of ventricular function in heart of various experimental groups. (B) Cumulative concentration-response curves of Ach induced relaxation in endothelium-intact aortic rings. (C) Cumulative concentration-response curves of SNP induced relaxation in endothelium-intact aortic rings. Values are expressed as mean ± SD. n = 6 per group. ^*^ *P*<0.05 vs control; ^#^ *P*<0.05 vs L-NAME.

The systolic contractility of the isolated heart was measured by the first temporal derivative of the left ventricular pressure (LVP) positive development (+dP/dt, in mmHg/s), and the isovolumetric relaxation was measured by the first temporal derivative of the LVP negative pressure (−dP/dt, in mmHg/s). In the heart of L-NAME rats, the rate of LV pressure rise (+dP/dt, in mmHg/s) and the rate of LV pressure decline (−dP/dt, in mmHg/s) was significantly (*P*<0.05) reduced. SA treatment significantly (*P*<0.05) promoted ventricular function in L-NAME rats (Fig. 4A). Sensitivity of the aortic rings to acetylcholine from rats given L-NAME was significantly (*P*<0.05) reduced. When aortic tissues from rats given SA were challenged with acetylcholine, the vasodilatation was almost restored (Fig. 4B). Exposure of the aortic rings to cumulative concentrations of nitrovasodilator, sodium nitroprusside produced dose-response curves that were almost superimposable, except for a minimally reduced relaxation in the aortic rings from rats given L-NAME (Fig. 4C).

### Differentially expressed mRNAs and protein

**Figure 5:**
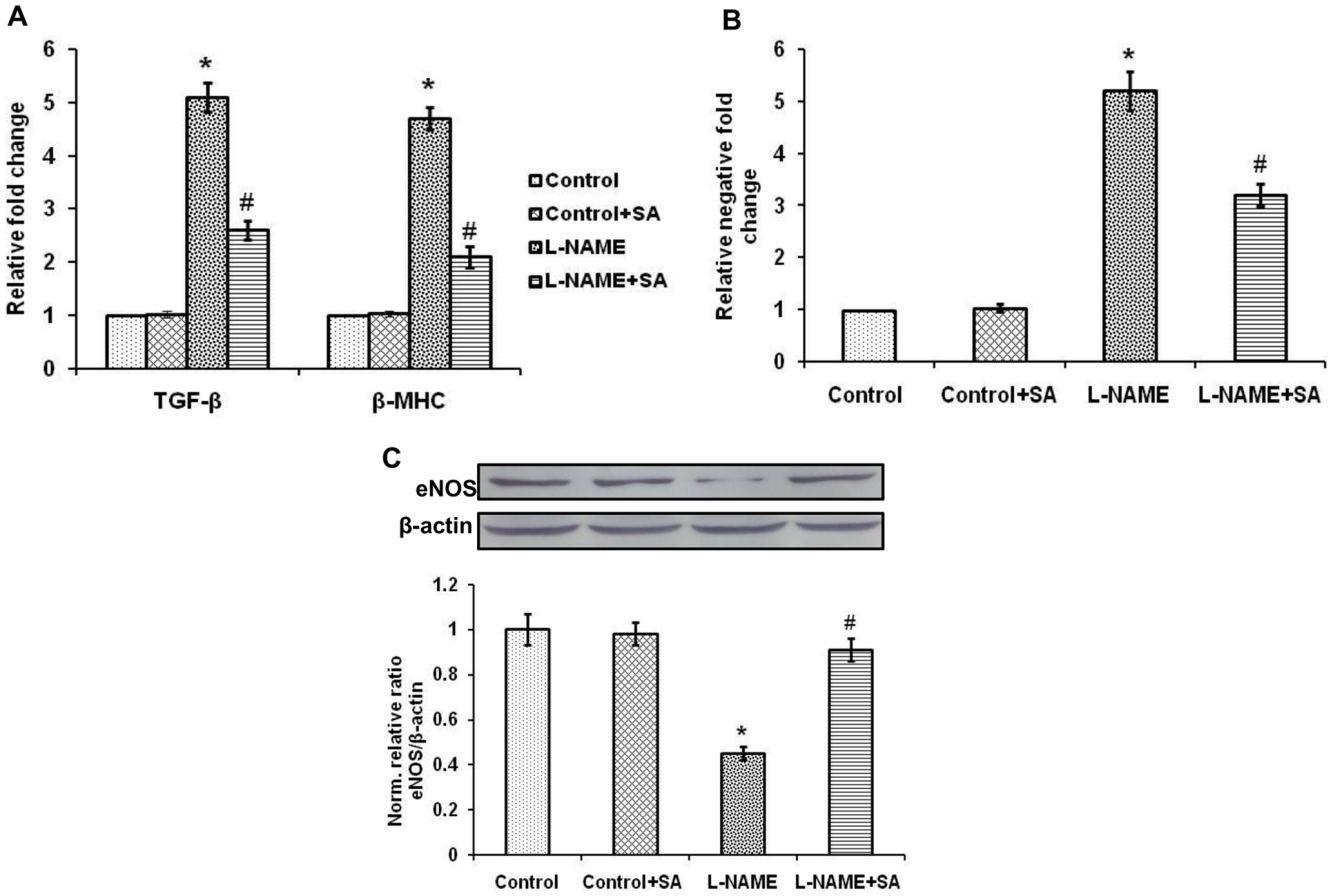
Sinapic acid prevents deregulated expression of cardiovascular genes. (A) Relative expression fold changes of TGF-β and β-MHC mRNAs in heart. (B) Relative expression negative fold change of eNOS mRNA in aorta. (C) Differential eNOS protein expression in aorta and its normalized value with β-actin. Values are expressed as means ± SD. All experiments were done in triplicates. ^*^ *P*<0.05 vs control; ^#^ *P*<0.05 vs L-NAME.

L-NAME rat hearts showed significantly (*P*<0.05) elevated mRNA expression of TGF-β and β-MHC, compared with control when normalized with GAPDH expression. SA treatment significantly (*P*<0.05) suppressed the expression of above mRNAs in L-NAME rat heart (Fig. 5A). eNOS mRNA and protein expressions in L-NAME aorta were significantly (*P*<0.05) decreased compared with control. SA treated aorta showed significantly increased eNOS expression compared with L-NAME (Fig. 5B and C, respectively).

### Oxidative stress and nitric oxide level – *in vitro* study

**Figure 6:**
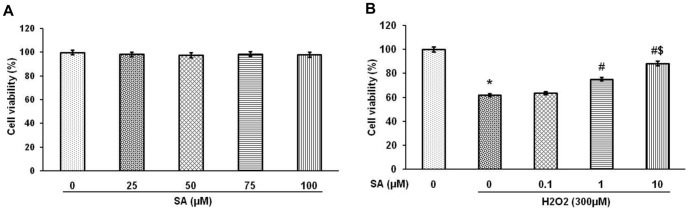
Sinapic acid protects EA.hy926 cells from H_2_O_2_-induced cytotoxicity. (A) The toxic effect of SA (25–100 µM) was measured in Ea.hy926 cells by MTT assay. (B) Different concentrations of SA (0.1–10 µM) were pre-treated for 24 h prior to incubation of cells with 300 µM of H_2_O_2_ for 4 h. MTT assay was performed and the values are expressed as mean ± SEM of three independent experiments. ^*^ *P*<0.05 vs Control; ^#^ *P*<0.05 vs H_2_O_2_ alone group; ^$^ *P*<0.05 vs 1 µM.

**Figure 7:**
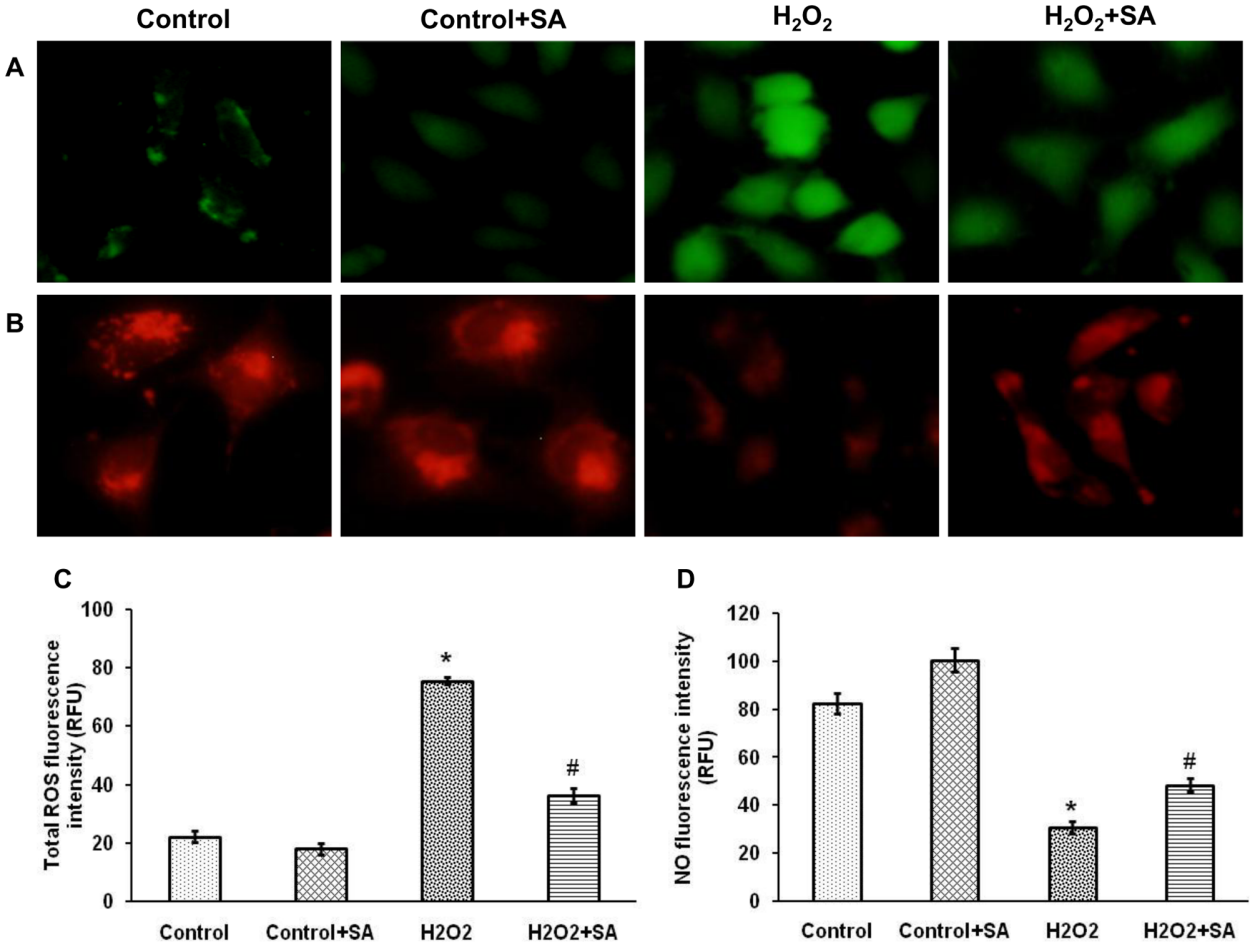
acid decreases total ROS and improves NO level in EA.hy926 cells. 10 µM SA was pre-treated for 24 h prior to incubation of cells with 300 µM of H_2_O_2_ for 4 h. (A) Intracellular total ROS level was measured by the fluorescent probe DCFH-DA and the images were obtained by fluorescence microscopy. (B) NO level was measured by the fluorescent probe DAR-4M AM and the images were obtained by fluorescence microscopy. The representative images from three independent experiments are shown. (C) Total ROS fluorescence intensity value was calculated using Adobe Photoshop version 7.0. (D) Fluorescence intensity measurement against NO was calculated using Adobe Photoshop version 7.0. RFU: Relative Fluorescence Unit. Values are expressed as mean ± SEM. ^*^ *P*<0.05 vs control; ^#^ *P*<0.05 vs H_2_O_2_.

The results of MTT assay explored that SA does not interfere with the viability of EA.hy926 cells upto 100 µM (Fig. 6A). Moreover, the cell viability was increased against H_2_O_2_-induced oxidative stress in a concentration dependent manner and among the three doses (0.1, 1 and 10 µM) 1 and 10 µM of SA have showed significant (*P*<0.05) effect when compared with H_2_O_2_ alone treated group (Fig. 6B). 10 µM showed significant (*P*<0.05) effect when compared with 1 µM, so we have chosen 10 µM dose for evaluation of total ROS and NO level. The elevation of oxidative stress upon H_2_O_2_ treatment and the amelioration effect of SA was shown by fluorescene analysis, which indicates SA significantly (*P*<0.05) protects cells from ROS generation (Fig. 7A and C). Further, NO level was also significantly (*P*<0.05) elevated in H_2_O_2_-induced cells upon SA treatment (Fig. 7B and D).

## Discussion

Oxidative stress via production of reactive oxygen species (ROS) plays an important role in the development of hypertension [37]. Endogenous nitric oxide (NO) regulates myocardial remodeling [38]. Chronic inhibition of NO produces volume-dependent elevation of blood pressure; and its physiological and pathological characteristics resemble essential hypertension. Besides, it is well established that acute inhibition of NO biosynthesis by *in vivo* administration of L-NAME, an L-arginine analog, leads to arterial hypertension and vasoconstriction [21]. In this study, L-NAME rats showed significantly increased systolic blood pressure and this increase was attenuated by sinapic acid (SA) treatment.

Free radical-scavenging enzymes such as SOD, CAT, and GPx are the first line of cellular defense against oxidative injury, decomposing superoxide and hydrogen peroxide, otherwise interacting to form the more reactive hydroxyl radical [39]. The equilibrium between the enzymatic antioxidants and free radicals is an important process for the effective removal of oxidative stress in intracellular organelles [40]. In this study, significantly lowered activities of the enzymes SOD, CAT and GPx were observed in heart and aorta of L-NAME rats. The decrease in the activities of SOD and CAT might be due to their increased utilization in scavenging ROS and their inactivation by excessive oxidants. GPx offers protection to the cellular and subcellular membranes from peroxidative damage by eliminating hydrogen peroxide and lipid peroxides and its declined activity may be due to the reduced availability of GSH [41]. Previous reports explored that oxidative stress derived from NADPH oxidase has been implicated in the physiopathogenesis of hypertensive LV remodeling [42], [43].

Lipid peroxidation, arising from the reaction of free radicals with lipids, has been linked with altered membrane structure and enzyme inactivation. Its end products measured as thiobarbituric acid reactive substances and lipid hydroperoxides were seen to be highly increased in heart and aortic tissues clearly indicating increased oxidative stress in L-NAME rats, which has been discussed above. SA attenuates oxidative stress by enhancing antioxidants and prevents membrane damage through its antioxidant efficiency was already reported [44]. Moreover, *in vitro* free radical scavenging effect of SA through its antioxidant potential was also already discovered [17], [18]. From these evidences, we come to a decision that the protective action of SA would be at least partially due to its antioxidant potential.

Cardiac hypertrophy correlates with increased blood pressure, increased fibrosis, collagen deposition, and reduced cardiac function. Hydroxyproline is a component of collagen and a quantitative index of fibrosis. Collagen accumulation occurs in the heart during heart failure and contributes to stiffening of the heart walls, impaired relaxation, impaired filling, and reduced cardiac output [45]. In this study, the elevated cardiac weight in hypertension was significantly suppressed by SA treatment, this may be due to the antihypertensive potential of SA which in turn reduces pressure load induced hypertrophy.

In the present study, sinapic acid with antioxidant function blunts L-NAME induced cardiovascular fibrosis as indicated by hydroxyproline level and histopathological analysis. A previous study indicated that oxidative stress generated during hypertensive condition has the potential to induce fibrosis [46]. Another study suggests that ROS production would contribute to the perivascular inflammation and subsequent myocardial fibrosis through angiotensin II (Ang II) [47]. In the current study, elevated ACE activity in hypertensive heart may enhance the conversion of angiotensin I (Ang I) into bioactive angiotensin II (Ang II) which clearly mirrors the part of Ang II in myocardial fibrosis. Moreover, cardiac oxidative stress promotes the development of cardiac fibrosis by upregulating TGF-β expression, which subsequently enhances cardiac collagen synthesis and suppresses collagen degradation in hypertensive rats [48].

In the current study, upregulated expression of TGF-β and β-MHC were observed in hypertensive condition. At the molecular level, pathological stresses induce multiple changes, including genetic reprogramming [49]. The upregulated gene TGF-β is a locally generated cytokine that has been implicated as a major contributor to tissue fibrosis and latest studies in humans and experimental models have shown increased myocardial TGF-β expression during cardiac hypertrophy and fibrosis [50], [51]. Upregulation of β-MHC transcription has been used as an early and sensitive marker of cardiac hypertrophy. In general, α-MHC is normally a predominant isoform occurring in heart and expression of β-MHC contributes to the overall poor functioning of the hypertrophic ventricle [52]. Recent studies provide evidence that the antioxidant apocynin attenuates oxidative stress and cardiac fibrosis in angiotensin II-induced cardiac diastolic dysfunction in mice [53]. Consistent with the prior study [44], this study also found SA with antioxidant function enhances the antioxidant network thereby it decreases the oxidative stress and fibrosis.

Cardiac contractile function impairment is one of the major pathogenic features of cardiac remodeling. The isolated heart Langendorff study explains that the hypertension induces ventricular dysfunction. It was previously known that, excess production and accumulation of extracellular matrix structural proteins, or fibrosis, results in enhanced stiffness of the myocardium and impedes ventricular contraction and relaxation, leading to distorted architecture and function of the heart. Excess collagen deposition and fibrosis has been clearly linked to myocardial stiffness, diastolic and systolic dysfunction [54], [55]. In the antioxidant point of view, Mapanga et al. explored that oleanolic acid, an antioxidant agent that blunts hyperglycemia-induced contractile dysfunction [56]. In consistent with the previous report, in this study L-NAME induction reduces the contractile function and SA treatment restores the ventricular function with its antioxidant property.

The protection of vascular function by SA was one of the significant findings of this study. Vascular endothelium plays a pivotal role in the pathophysiology of cardiovascular system. Nitric oxide (NO), generated by eNOS, is physiologically important in vascular homeostasis, which readily activates guanylyl cyclase and increases cyclic GMP formation in vascular smooth muscle [57]. Endogenous NO reacts with superoxide to form peroxynitrite (ONOO^−^), which is capable of either oxidizing or nitrating various biological substrates [58]. Previous study illustrates that, pioglitazone administration reduced oxidative stress, prevented breakdown of NO and increased NO levels, thereby restoring the endothelial function in aorta of diabetic rat [30]. In the current study, decreased response of aorta to Ach induced endothelium dependent relaxations occurred in L-NAME induced hypertensive rats might be due to the reduced production/bioavailability of NO and elevated oxidative stress. Moreover, in the present study eNOS mRNA and protein expressions were down regulated coupled with increased ACE activity in the aorta of L-NAME rats whereas SA restored the above. In a similar study, L-NAME treated rats showed definite increase in systolic blood pressure, decrease in eNOS gene expression in aortic tissue and decreased response of aorta to acetylcholine. Further, ACE inhibition by enalapril or quinapril was equally effective in improving endothelial vasodilator function and restoring aortic eNOS mRNA [59]. Likewise, protective action of SA might be due to increased NO level and decreased ACE activity through antioxidant potential, which was strongly supported by our *in vitro* endothelial cell culture study.

In order to support our *in vivo* results obtained from isolated aortic ring experiments in organ bath system, we analyzed the protective effect of SA on endothelial cell injury induced by H_2_O_2_ *in vitro*. In this study, we demonstrated that H_2_O_2_ could markedly increase cell death, induce oxidative stress and decline NO production. The up-regulation of ROS in vascular lesions will exert detrimental effects on the peroxidation of membrane lipids, endothelium-derived enzyme inactivation, apoptotic occurrences, etc [60]. In contrast, antioxidants/agents that react preferentially with ROS to inactivate them or enhance cellular antioxidant defenses can protect cells from the damaging effects of oxygen radicals [61]. Observations of a former study on the current model have provide preliminary evidence that antioxidant TSN IIA protects EA.hy926 cells against H_2_O_2_ damage, which is mainly associated with the ROS generation [35]. Similarly, our study showed that SA not only reduced the intracellular ROS induced by H_2_O_2_, but also effectively increase cell viability and NO level in H_2_O_2_-induced endothelial cells, which shows the protective potential of SA on endothelial cells.

In conclusion, hypertensive rats treated with SA demonstrated attenuated hypertension and improved cardiovascular function. SA treatment reduced the oxidative stress and myocardial fibrosis observed in hypertensive rats. These findings suggest that SA may have great therapeutic potential in the treatment of hypertensive heart disease.

## References

[1] Lawes CM, Vander Hoorn S, Rodgers A (2008) Global burden of blood-pressure-related disease, 2001. Lancet 371:1513–1518. 18456100 10.1016/S0140-6736(08)60655-8

[2] Zhang Y, Shao L, Ma A, Guan G, Wang J, et al (2014) Telmisartan delays myocardial fibrosis in rats with hypertensive left ventricular hypertrophy by TGF-β1/Smad signal pathway. Hypertens Res 37:43–49. 24089264 10.1038/hr.2013.119

[3] Cohn JN, Ferrari R, Sharpe N (2000) Cardiac remodeling—concepts and clinical implications: a consensus paper from an international forum on cardiac remodeling. Behalf of an International Forum on Cardiac Remodeling. J Am Coll Cardiol 35:569–582. 10716457 10.1016/s0735-1097(99)00630-0

[4] Galderisi M, de Divitiis O (2008) Risk Factor-induced Cardiovascular Remodeling and the Effects of Angiotensin-Converting Enzyme Inhibitors. J Cardiovasc Pharmacol 51:523–531. 18520954 10.1097/FJC.0b013e31817751a7

[5] Kehat I, Molkentin JD (2010) Molecular Pathways Underlying Cardiac Remodeling During Pathophysiological Stimulation. Circulation 122:2727–2735. 21173361 10.1161/CIRCULATIONAHA.110.942268 PMC3076218

[6] Cataliotti A, Tonne JM, Bellavia D, Martin FL, Oehler EA, et al (2011) Longterm cardiac pro-B-type natriuretic peptide gene delivery prevents the development of hypertensive heart disease in spontaneously hypertensive rats. Circulation 123:1297–1305. 21403100 10.1161/CIRCULATIONAHA.110.981720 PMC3081597

[7] Becker LB (2004) New concepts in reactive oxygen species and cardiovascular reperfusion physiology. Cardiovasc Res 61:461–470. 14962477 10.1016/j.cardiores.2003.10.025

[8] Juránek I, Bezek S (2005) Controversy of free radical hypothesis: reactive oxygen species–cause or consequence of tissue injury? Gen Physiol Biophys 24:263–278. 16308423

[9] Sabri A, Hughie HH, Lucchesi PA (2003) Regulation of hypertrophic and apoptotic signaling pathways by reactive oxygen species in cardiac myocytes. Antioxid Redox Signal 5:731–740. 14588146 10.1089/152308603770380034

[10] Taniyama Y, Griendling KK (2003) Reactive oxygen species in the vasculature: molecular and cellular mechanisms. Hypertension 42:1075–1081. 14581295 10.1161/01.HYP.0000100443.09293.4F

[11] Paravicini TM, Touyz RM (2008) NADPH oxidases, reactive oxygen species, and hypertension: clinical implications and therapeutic possibilities. Diabetes Care 31 Suppl 2S170–S180. 18227481 10.2337/dc08-s247

[12] Matsushita K, Morrell CN, Cambien B, Yang SX, Yamakuchi M, et al (2003) Nitric oxide regulates exocytosis by S-nitrosylation of N-ethylmaleimide-sensitive factor. Cell 115:139–150. 14567912 10.1016/s0092-8674(03)00803-1 PMC2846406

[13] Morrell CN, Matsushita K, Chiles K, Scharpf RB, Yamakuchi M, et al (2005) Regulation of platelet granule exocytosis by S-nitrosylation. Proc Natl Acad Sci U S A 102:3782–3787. 15738422 10.1073/pnas.0408310102 PMC553307

[14] Attia DM, Verhagen AM, Stroes ES, van Faassen EE, Gröne HJ, et al (2001) Vitamin E alleviates renal injury, but not hypertension, during chronic nitric oxide synthase inhibition in rats. J Am Soc Nephrol 12:2585–2593. 11729226 10.1681/ASN.V12122585

[15] Kumar S, Saravanakumar M, Raja B (2010) Efficacy of piperine, an alkaloidal constituent of pepper on nitric oxide, antioxidants and lipid peroxidation markers in L-NAME induced hypertensive rats. Int J Res Pharm Sci 1:300–307.

[16] Andreasen MF, Landbo AK, Christensen LP, Hansen A, Meyer AS (2001) Antioxidant effects of phenolic rye (Secale cereale L.) extracts, monomeric hydroxycinnamates and ferulic acid dehydrodimers on human low-density lipoproteins. J Agric Food Chem 49:4090–4096. 11513715 10.1021/jf0101758

[17] Roy SJ, Prince PSM (2012) Protective effects of sinapic acid on lysosomal dysfunction in isoproterenol induced myocardial infarcted rats. Food Chem Toxicol 50:3984–3989. 22921837 10.1016/j.fct.2012.08.017

[18] Roy SJ, Prince PSM (2013) Protective effects of sinapic acid on cardiac hypertrophy, dyslipidaemia and altered electrocardiogram inisoproterenol-induced myocardial infarcted rats. Eur J Pharmacol 699:213–218. 23178800 10.1016/j.ejphar.2012.11.012

[19] Kanchana G, Shyni WJ, Rajadurai M, Periasamy R (2011) Evaluation of Antihyperglycemic Effect of Sinapic Acid in Normal and Streptozotocin-Induced Diabetes in Albino Rats. Global Journal of Pharmacology 5:33–39.

[20] Zou Y, Kim AR, Kim JE, Choi JS, Chung HY (2002) Peroxynitrite scavenging activity of sinapic acid (3,5-dimethoxy-4-hydroxycinnamic acid) isolated from Brassica juncea. J Agric Food Chem 50:5884–5890. 12358454 10.1021/jf020496z

[21] Saravanakumar M, Raja B (2011) Veratric acid, a phenolic acid attenuates blood pressure and oxidative stress in L-NAME induced hypertensive rats. Eur J Pharmacol 671:87–94. 21937012 10.1016/j.ejphar.2011.08.052

[22] Susic D, Varagic J, Ahn J, Matavelli L, Frohlich ED (2007) Long-term mineralocorticoid receptor blockade reduces fibrosis and improves cardiac performance and coronary hemodynamics in elderly SHR. Am J Physiol Heart Circ Physiol 292:H175–H179. 16905598 10.1152/ajpheart.00660.2006

[23] Kakkar P, Das B, Viswanathan PN (1984) A modified spectrophotometric assay of superoxide dismutase. Indian J Biochem Biophys 21:130–132. 6490072

[24] Sinha AK (1972) Colorimetric assay of catalase. Anal Biochem 47:389–394. 4556490 10.1016/0003-2697(72)90132-7

[25] Rotruck JT, Pope AL, Ganther HE, Swanson AB, Hafeman DG, et al (1973) Selenium: biochemical role as a component of glutathione peroxidise. Science 179:588–590. 4686466 10.1126/science.179.4073.588

[26] Ellman GL (1959) Tissue sulfhydryl groups. Arch Biochem Biophys 82:70–77. 13650640 10.1016/0003-9861(59)90090-6

[27] Lowry OH, Rosebrough NJ, Farr AL, Randall AL (1951) Protein measurement with the Folin phenol reagent. J Biol Chem 193:265–275. 14907713

[28] Niehaus WG, Samuelsson B (1968) Formation of malondialdehyde from phospholipid arachidonate during microsomal lipid peroxidation. Eur J Biochem 6:126–130. 4387188 10.1111/j.1432-1033.1968.tb00428.x

[29] Jiang ZY, Hunt JV, Wolff SP (1992) Ferrous ion oxidation in the presence of xylenol orange for detection of lipid hydroperoxide in low density lipoprotein. Anal Biochem 202:384–389. 1519766 10.1016/0003-2697(92)90122-n

[30] Majithiya JB, Paramar AN, Balaraman R (2005) Pioglitazone, a PPAR γ agonist, restores endothelial function in aorta of streptozotocin-induced diabetic rats. Cardiovasc Res 66:150–161. 15769458 10.1016/j.cardiores.2004.12.025

[31] Sharma DK, Manral A, Saini V, Singh A, Srinivasan BP, et al (2012) Novel diallyldisulfide analogs ameliorate cardiovascular remodeling in rats with L-NAME-induced hypertension. Eur J Pharmacol 691:198–208. 22819707 10.1016/j.ejphar.2012.07.022

[32] Fenning A, Harrison G, Rose'meyer R, Hoey A, Brown L (2005) L-arginine attenuates cardiovascular impairment in DOCA-salt hypertensive rats. Am J Physiol Heart Circ Physiol 289:H1408–H1416. 15923320 10.1152/ajpheart.00140.2005

[33] Sukumaran SV, Singh TU, Parida S, Narasimha Reddy ChE, Thangamalai R, et al (2013) TRPV4 channel activation leads to endothelium-dependent relaxation mediated by nitric oxide and endothelium-derived hyperpolarizing factor in rat pulmonary artery. Pharmacol Res 78:18–27. 24075884 10.1016/j.phrs.2013.09.005

[34] Schmittgen TD, Livak KJ (2008) Analyzing real-time PCR data by the comparative CT method. Nat Protoc 3:1101–1108. 18546601 10.1038/nprot.2008.73

[35] Jia LQ, Yang GL, Ren L, Chen WN, Feng JY, et al (2012) Tanshinone IIA reduces apoptosis induced by hydrogen peroxide in the human endothelium-derived EA.hy926 cells. J Ethnopharmacol 143:100–108. 22750433 10.1016/j.jep.2012.06.007

[36] Siamwala JH, Dias PM, Majumder S, Joshi MK, Sinkar VP, et al (2013) L-theanine promotes nitric oxide production in endothelial cells through eNOS phosphorylation. J Nutr Biochem 24:595–605. 22819553 10.1016/j.jnutbio.2012.02.016

[37] Dikalov SI, Ungvari Z (2013) Role of mitochondrial oxidative stress in hypertension. Am J Physiol Heart Circ Physiol 305 : H1417– H1427. 10.1152/ajpheart.00089.2013 PMC3840266 24043248

[38] Kazakov A, Hall R, Jagoda P, Bachelier K, Müller-Best P, et al (2013) Inhibition of endothelial nitric oxide synthase induces and enhances myocardial fibrosis. Cardiovasc Res 100:211–221. 23863197 10.1093/cvr/cvt181

[39] Rodrigo R, Libuy M, Feliú F, Hasson D (2013) Oxidative stress-related biomarkers in essential hypertension and ischemia-reperfusion myocardial damage. Dis Markers 35:773–790. 24347798 10.1155/2013/974358 PMC3856219

[40] Senthil S, Veerappan RM, Ramakrishna Rao M, Pugalendi KV (2004) Oxidative stress and antioxidants in patients with cardiogenic shock complicating acute myocardial infarction. Clin Chim Acta 348:131–137. 15369746 10.1016/j.cccn.2004.05.004

[41] Li H, Xie YH, Yang Q, Wang SW, Zhang BL, et al (2012) Cardioprotective effect of paeonol and danshensu combination on isoproterenol-induced myocardial injury in rats. PLoS ONE 7:e48872. 23139821 10.1371/journal.pone.0048872 PMC3490947

[42] Maytin M, Siwik DA, Ito M, Xiao L, Sawyer DB, et al (2004) Pressure overload-induced myocardial hypertrophy in mice does not require gp91phox. Circulation 109:1168–1171. 14981002 10.1161/01.CIR.0000117229.60628.2F

[43] Murdoch CE, Zhang M, Cave AC, Shah AM (2006) NADPH oxidase-dependent redox signalling in cardiac hypertrophy, remodelling and failure. Cardiovasc Res 71:208–215. 16631149 10.1016/j.cardiores.2006.03.016

[44] Pari L, Jalaludeen AM (2011) Protective role of sinapic acid against arsenic – Induced toxicity in rats. Chem Biol Interact 194:40–47. 21864513 10.1016/j.cbi.2011.08.004

[45] Seymour EM, Singer AM, Bennink MR, Parikh RV, Kirakosyan A, et al (2008) chronic intake of a phytochemical-enriched diet reduces cardiac fibrosis and diastolic dysfunction caused by prolonged salt-sensitive hypertension. J Gerontol A Biol Sci Med Sci 63:1034–1042. 18948553 10.1093/gerona/63.10.1034 PMC2640469

[46] Hiremath P, Bauer M, Aguirre AD, Cheng HW, Unno K, et al (2014) Identifying early changes in myocardial microstructure in hypertensive heart disease. PLoS One 9:e97424. 24831515 10.1371/journal.pone.0097424 PMC4022613

[47] Kai H, Mori T, Tokuda K, Takayama N, Tahara N, et al (2006) Pressure overload–induced transient oxidative stress mediates perivascular inflammation and cardiac fibrosis through angiotensin II. Hypertens Res 29:711–718. 17249527 10.1291/hypres.29.711

[48] Zhao W, Zhao T, Chen Y, Ahokas RA, Sun Y (2008) Oxidative stress mediates cardiac fibrosis by enhancing transforming growth factor-beta1 in hypertensive rats. Mol Cell Biochem 317:43–50. 18581202 10.1007/s11010-008-9803-8

[49] Pandya K, Smithies O (2011) β-MyHC and Cardiac Hypertrophy: size does matter. Circ Res 109:609–610. 21885833 10.1161/CIRCRESAHA.111.252619

[50] Rosenkranz S (2004) TGF-β1 and angiotensin networking in cardiac remodeling. Cardiovasc Res 63:423–432. 15276467 10.1016/j.cardiores.2004.04.030

[51] Boluyt MO, O'Neill L, Meredith AL, Bing OH, Brooks WW, et al (1994) Alterations in cardiac gene expression during the transition from stable hypertrophy to heart failure: Marked upregulation of genes encoding extracellular matrix components. Circ Res 75:23–32. 8013079 10.1161/01.res.75.1.23

[52] Krenz M, Robbins J (2004) Impact of beta-myosin heavy chain expression on cardiac function during stress. J Am Coll Cardiol 44:2390–2397. 15607403 10.1016/j.jacc.2004.09.044

[53] Li YQ, Li XB, Guo SJ, Chu SL, Gao PJ, et al (2013) Apocynin attenuates oxidative stress and cardiac fibrosis in angiotensin II-induced cardiac diastolic dysfunction in mice. Acta Pharmacol Sin 34:352–359. 23334241 10.1038/aps.2012.164 PMC4002490

[54] Diez J, Querejeta R, Lopez B, Gonzalez A, Larman M, et al (2002) Losartan-dependent regression of myocardial fibrosis is associated with reduction of left ventricular chamber stiffness in hypertensive patients. Circulation 105:2512–2517. 12034658 10.1161/01.cir.0000017264.66561.3d

[55] Manabe I, Shindo T, Nagai R (2002) Gene expression in fibroblasts and fibrosis: involvement in cardiac hypertrophy. Circ Res 91:1103–1113. 12480810 10.1161/01.res.0000046452.67724.b8

[56] Mapanga RF, Rajamani U, Dlamini N, Zungu-Edmondson M, Kelly-Laubscher R, et al (2012) Oleanolic acid: a novel cardioprotective agent that blunts hyperglycemia-induced contractile dysfunction. PLoS One 7:e47322. 23091615 10.1371/journal.pone.0047322 PMC3473042

[57] Tawfik HE, Cena J, Schulz R, Kaufman S (2008) Role of oxidative stress in multiparity-induced endothelial dysfunction. Am J Physiol Heart Circ Physiol 295:H1736–H1742. 18757488 10.1152/ajpheart.87.2008

[58] Zobali F, Cakici I, Karasu C (2001) Effects of peroxynitrite on the reactivity of diabetic rat aorta. Pharmacology 63:58–64. 11408833 10.1159/000056113

[59] De Gennaro Colonna V, Rossoni G, Rigamonti A, Bonomo S, Manfredi B, et al (2002) Enalapril and quinapril improve endothelial vasodilator function and aortic eNOS gene expression in L-NAME-treated rats. Eur J Pharmacol 450:61–66. 12176110 10.1016/s0014-2999(02)02046-0

[60] Xu HB, Huang ZQ (2007) Icariin enhances endothelial nitric-oxide synthase expression on human endothelial cells in vitro. Vascul Pharmacol 47:18–24. 17499557 10.1016/j.vph.2007.03.002

[61] Fang WT, Li HJ, Zhou LS (2010) Protective effects of prostaglandin E1 on human umbilical vein endothelial cell injury induced by hydrogen peroxide. Acta Pharmacol Sin 31:485–492. 20305680 10.1038/aps.2010.23 PMC4007667

